# Human Papillomavirus associated prevention: knowledge, attitudes, and perceived risks among men who have sex with men and transgender women in Pakistan: a qualitative study

**DOI:** 10.1186/s12889-022-12775-z

**Published:** 2022-02-22

**Authors:** Muslima Ejaz, Anna Mia Ekström, Alyan Ahmed, Aymen Haroon, Dania Ali, Tazeen Saeed Ali, Mariano Salazar

**Affiliations:** 1 Department of Global Public Health Karolinska Institutet, Stockholm, Sweden; 2 Department of Community Health Sciences, the Aga Khan University, Karachi, Pakistan; 3 Department of Biostatistics and Epidemiology, School of Public Health, Boston University, Boston, Massachusetts United States; 4 School of Nursing, the Aga Khan University, Karachi, Pakistan

**Keywords:** HPV prevention, Risk perceptions, HPV knowledge, Sexual and gender minority, MSM, Transgender women, Qualitative analysis, Pakistan

## Abstract

**Objectives:**

Men who have sex with men (MSM) and transgender individuals are at higher risk of genital warts and anal cancer due to sexually transmitted human papillomavirus infection. This study explores MSM and transgender women’s perceptions of Human papillomavirus (HPV) infection and HPV prevention strategies (screening and vaccination) in Pakistan.

**Design:**

A qualitative study using focus group discussions (FGD) with self-identified MSM, male sex workers and transgender women were conducted between March 2019 to August 2019 in Karachi, Pakistan.

**Methods:**

Participants were recruited from community-based organization (CBO) working for MSM and transgender women. A total of 38 men and 10 transgender women took part in 6 FGDs. Discussions were recorded, translated, transcribed verbatim and analyzed using content analysis.

**Results:**

Three themes were identified from the emerging analysis. These are, 1) Knowledge and risk perceptions about STIs and HPV, 2) Beliefs and attitudes towards HPV prevention, 3) Participant’s recommendations for HPV vaccination and anal Pap screening. Participants described lack of knowledge of HPV and its health consequences as HIV is the only focus of attention of the government and the local CBOs. None of participants had heard about HPV prevention including vaccination and anal Pap screening for men but expressed a positive attitude towards prevention. Genital warts and anal cancer were perceived as severe potential consequences of a known risk behaviors. All participants stated they would be interested in taking an HPV vaccine but acknowledged that the provision of services for sexually transmitted infections (STI) are inadequate to meet the needs of key populations and are not prioritized by the government. The main perceived barriers to access HPV prevention included cost and challenges to access public health care services or openly discussing one’s sexual orientation with health care providers. Participants generally preferred the CBO for more professional, unbiased staff attitudes that respect patients’ integrity, confidentiality and privacy. Most participants thought that in case the government is non-cooperative, CBOs should work in the interest of HPV eradication and generate funds through international funding.

**Conclusions:**

The findings from this study can help public health policy and researchers to understand this minority’s perspective on HPV prevention. Given the low level of knowledge about HPV infection and its negative health consequences there is a need of HPV education combined with STI education and awareness through HPV brochures to educate the target population effectively.

**Supplementary Information:**

The online version contains supplementary material available at 10.1186/s12889-022-12775-z.

## Background

Human papillomavirus (HPV) infection is the most common viral sexually transmitted infection (STI) worldwide [1] and classified as low-risk and high-risk HPV types depending on their oncogenic potential [2]. HPV has a causal role in both benign (ano-genital warts) and malignant lesions (penile, anal, and oropharyngeal cancers) [3]. Men who have sex with men (MSM) are particularly prone to STI given their high-risk sexual behaviors such as engaging in group sex, having large number of sexual partners and practicing condom-less receptive anal sex [4]. Moreover, anal HPV infection is worsened by HIV related immunosuppression [5]. A recent meta-analysis has reported a pooled prevalence of anal HPV infection of 63.9% and 92.6% for HIV uninfected and infected MSM respectively [6].

Persistent infection with high-risk HPV type most notably HPV type 16 is the major cause of anal cancer [7] and has been detected in 80.7% of anal cancer [8, 9]. The reported global incidence of anal cancer in MSM is up to 35 cases per 100,000 [10] compared to just 1 case per 100,000 heterosexual males and even up to 131/100,000 in HIV infected MSM [11]. Such disparities result from the prevalence and incidence of HPV infection that is much higher in MSM [12–14] when compared with the general population [15–17] and even greater in HIV infected MSM when compared to non-HIV infected MSM (98% verses 57%) [18–20].

The most effective strategy available to prevent HPV and related diseases in heterosexual and MSM populations is prophylactic vaccination. The Advisory Committee on Immunization Practices recommends vaccinating all MSM up through age of 26 years due to the increased risk of anal cancer in this key population [21] A recent meta-analysis [22] has reported the average HPV vaccine acceptability among MSM of 63% with the highest from China (97%) [23] and the lowest from Australia (30%) [24], however an increasing trend of vaccine uptake is being reported by others over time [25]. Studies have reported decline in HPV associated anogenital warts among MSM since the introduction of HPV vaccination program [26, 27].

Furthermore, anal Papanicolaou (Pap) screening for the precancerous HPV anal lesions as a secondary prevention may have greater utility in preventing future anal cancer [28–31] and indeed is a cost-effective method for anal cancer prevention among MSM [32, 33]. Anal cancer screening could follow a very similar process to that for cervical cancer screening, however, with an exclusive focus on high-risk groups [34].

HPV vaccination is limited in Pakistan. HPV vaccine is available to private health care providers and pharmacies but is not yet part of national immunization programs targeting heterosexual or Lesbian, Gay, Bisexuals, Transgender and Queer (LGBTQ) populations. Before establishing vaccination and screening programs targeting LGBTQ populations, the health system needs to assess what factors exist that can hinder or facilitate the implementation of such programs among sexual and gender minorities.

Previous studies done in Pakistan on HPV vaccine knowledge, attitudes and barriers among adults have found the low knowledge among men, moreover exorbitant cost, inaccessibility and misconceptions around vaccination were the barriers [35, 36] These studies have provided important information to action however none has focused on sexual and gender minorities which as described before are more at risk. Thus, our study aims to explore Pakistani MSM and transgender women’s knowledge on HPV infection, screening and vaccination services and to identify barriers and facilitators among Pakistani MSM and transgender women for screening and vaccination against HPV.

### Theoretical framework

Our exploratory research question was firmly guided by a theory of planned behavior (TPB). This theory stated that *attitudes and beliefs* are meaningful predictors of human behavior [37, 38], Thus, an individual suffering a particular disease evaluates his/her susceptibility to the problem, the severity of it and its social and physical consequences to decide on a course of action (or not). We used TPB theory to identify the topics to explore during data collection including attitudes (positive or negative), subjective norms (a person’s perception about a specific behavior influenced by the opinions of others) and control beliefs (a person’s perception of factors facilitating/hindering action) towards HPV vaccination and screening.

## Methods

### Study design

A qualitative exploratory study.

### Study setting

Pakistan is an Islamic republic country, where culture, society, and law incorporate religion in all codes and values that determine everyday life of a common person. Under the Islamic belief sex outside marriage, including sex with the same sex, is considered, offensive and taboo [39]. In Pakistan, sexual minorities are the target of discrimination and exclusion at all societal levels [40]. However, on physical and mental health grounds, a recent recognition of MSM as a vulnerable group is a highly commendable aspect of the Pakistan’s government’s policy and program. Several community-based organizations (CBO) and the Pakistani government are now investing efforts into establishing various health services for these high-risk and vulnerable groups including gays, male sex workers, transgender women and injecting drug users.

This study was performed in a sexual health clinic being run with a name of “Male Sexual Health” for MSM and transgender women by a CBO, Perwaaz Trust in Karachi Pakistan. This CBO provides care and support to the target population through the project team of outreach workers and counselors. Participation was offered to MSM and transgender women coming to this sexual health clinic.

### Participants and sampling procedures

The target population for this research was sexually active transgender women and MSM of vaccine eligible age from 18 to 26 years.

### Sampling and recruitment

Maximum variation sampling was used to recruit the informants. We aimed to select participants who range widely on the dimension of interest i.e., the sexual and gender identity, for instance, ***MSM*** who are in relationships but outwardly closeted, ***male sex workers (MSW)*** who are males and undertakes receptive anal or oral sexual activity with a man in return of money or other financial benefits, and ***transgender women*** or ***“HIJRA”*** and bisexuals who have sex with men as well as with women [39, 40].

The principal investigator (ME) and her team (AA,AH,DA) created a recruitment leaflet that included the topic and rationale of the study, the procedure of the study, participant inclusion criteria, information about the reimbursement for the transport cost and the willingness to give informed consent. That leaflet was disseminated to the target population through Parwaaz CBO, other CBOs and the National AIDS Control Program that provide services.

### Data Collection

Data collection was conducted between March to August 2019 by principal investigator (ME). Six focus group discussions (FGDs) (8 to 9 participants in each, 48 people in total) were conducted until data saturation was reached [41, 42]. FGDs were grouped by age and included MSM, male sex workers, bisexuals and transgender women. Overall, they lasted between 1.5 and 2 h. FGDs were audio-recorded and transcribed verbatim.

### Instrument development

A semi-structured interview guide was created based on the theory of planned behavior and a literature review. Open-ended questions with their relevant probes were used to elicit perceptions and beliefs towards their own susceptibility for HPV infection, attitudes towards HPV vaccination and regular anal Pap screening, HPV knowledge and community factors influencing vaccination attitudes. New topics arising during the FGDs were added to the FGD guide and explored with the next group. The FGD guide was shared with a panel of qualitative researcher and people from the LGBTQ community who provided feedback on the questions clarity and wordings.

### Use of Vignette in our study

Since HPV and sexual and gender minority is extremely under researched topic and underserved population in our country, it was assumed that MSM and transgender women would have very little context and working knowledge in which to understand HPV infection, its sequala and vaccination and other preventive services against HPV infection; therefore, the vignette was prepared by PI (ME). Vignettes in qualitative research are a kind of short stories or topic related scenarios inclusive of text or image that provide a context to the study participants to which responses are asked [43] to explore attitudes, beliefs and perceptions regarding sensitive issues [44, 45]. The principal investigator (ME) incorporated the following points into the vignette (1) key information about HPV; (2) anogenital warts—to increase perceived threat of HPV infection; and (3) alternative settings for vaccination and anal Pap-screening were explored.

### Analysis

Qualitative content analysis [46] was used to identify the manifest and latent content of the data. First, an open and an in-vivo coding process was used to analyze the data. Then, a focused coding process was used to code the data according to the theoretical frameworks guided by the topic schedule to reflect the scope of our inquiry. In the next step, codes were grouped into categories, and categories into subthemes and then themes. The team of ME (PI) and AA, AH, and DA coded the 3 FGDs together to ensure that the information was similarly coded. To enhance the trustworthiness of the findings, remaining three transcripts were hand-coded separately, and then the team met to compare the codes, resolve any coding discrepancies, and finally discuss the codebook. ME and MS finalized the sub themes and themes. All quotations are drawn from the focus group participants.

### Study Sample description/ Sample characteristics

A total of 48 participants took part in 6 focus group discussions. Most of the participants identified as gay and all had been sexually active with their male partners. The median (IQR) age was 22.5 (20–25) with almost 30% ≤ 20 years of age, most of the participants were poor with the minimum monthly income of just PKR 5,000 which is equivalent to 30 USD. Almost 40% of study participants were sex workers (19 of 48), 42% had no education, A total of 10 participants were transgender women. Two of the participants self-reported to be HIV positive, and HIV infection was central to their experience with the healthcare system and consequently their subsequent view on their HPV prevention (Table 1).

**Table 1:** Sociodemographic characteristics of study participants from sexual and gender minorities of Pakistan (*n* = 48)

**Age mean (± SD)**	22.4 (± 2.7)
**Education n (%)**	
None	20 (41.7)
Primary	10 (20.8)
Secondary	10 (20.8)
Intermediate	3 (6.2)
Graduate	3 (6.2)
Master students	2 (4.3)
Income median (IQR)*	12,000 (10,000–17,250)
**Age at first intercourse m(± SD)**	15.13(± 3.8)
**Sexual orientation n (%)**	
Homosexual	29 (60.4)
Bisexual	9(18.7)
Transgender women	10 (20.9)
**Sex workers n (%)**	19(39.5)
**HIV status n (%)**	
HIV positive	2(4.2)
HIV negative	46(95.8)
**Preferred anal sex role n (%)**	
Mainly receptive	42(87.5)
Mainly insertive	8 (12.5)
**Condom Use n (%)**	
Consistent	4(8.3)
Inconsistent	20 (41.7)
Never	24 (50)

*Inter quartile range

### Ethical considerations

The Human Research Ethics Committee of the Aga Khan (3612 – CHS- ERC – 15) approved this study as well as the informed consent procedure. Participants were provided (also read by ME) with an information sheet that described the purpose of the study and contained the primary investigator’s contact information. The investigator reviewed the informed consent form with each participant and notified them of their right to withdraw from the study at any time. Written consent in the form of signature or thumb impression was obtained prior to data collection.

The interviews were conducted in a private meeting room of a CBO and the participants were assured about the confidentiality and anonymity of the collected data and were informed about their free will to be part of the discussion, moreover, they were explained that there would be no effect on their service access if they were denied participation. All participants were offered PKR 200 (Equivalent to 1.25US$) to reimburse transport costs.

## Results

The main findings from the focus group discussions from this study were categorized into three themes and their sub-themes (Table 2). The perceptions and beliefs expressed in each of these subthemes are represented by the selected quotes taken from the discussion transcripts.

**Table 2:** Themes and sub themes of the study

Themes	Subthemes
Knowledge and risk perceptions about STIs and HPV	Limited Knowledge about STIs and HPV
	Low risk-perception to HPV infection and anal cancer
Beliefs and attitudes towards HPV prevention (vaccination and regular anal Pap screening)	Positive attitudes towards HPV vaccination and screening
	Barriers for HPV vaccination and screening
Participants recommendations for HPV vaccination and anal Pap screening	Need to raise awareness about HPV and its prevention
	Training of Health Care Providers
	Integration of HPV Prevention Service delivery

## Theme 1: Knowledge and risk perceptions about STI and HPV

### Limited knowledge about STIs and HPV

Our study participants had a limited knowledge on STIs and HPV with their knowledge being mainly on HIV prevention. They reflected that although they follow several sources of information (online websites, friends, family doctor and doctors in CBO) the focus of these sources was more on HIV symptoms and prevention than on other STIs. HPV awareness was very poor and in general this was a new concept to them.

*‘‘HIV is one of the most talked about sexually transmitted diseases in our [referring to MSM] community, considering it as the most common STI, but then after knowing that HPV is the most actual common STI maybe I should prevent myself from getting it”* (FGD # 5, 26-year-old, MSM).

This limited knowledge translated into poor recognition of STIs symptoms. STIs symptoms were sometimes misunderstood by study participants as generated by other causes such as weather.

*“[LGBTQ people] think having itching, burning, redness of skin in sensitive areas is because of hot weather [We] can’t appreciate having gonorrhea – for example”.* (FGD # 1, 22 year old male sex worker).

During the FGDs they acknowledged that certain sexual practices put them at risk of contracting STIs. For example, they identified that having multiples sexual partners, sex with an HIV positive partner, sex work and condom less sex due to avoiding missing sexual encounters or due to fearing having less pleasure were sexual risk-taking behaviors among their community.

*“We are more adventurous when it comes to sex and [we] follow multiple routes, pleasure matters to [us], opportunity matters to [us]”.* (FGD # 3, 19-years-old MSM).

*“Vulnerability of a sex worker for STI is due to his sexual attitude – [We] do sex with multiple men, 4 to 5 times in a day, obviously, we are at risk no matter how much safe we make it. [We] are always at risk”. As sex workers, we are exposed to HPV more, clients do not use condoms, we [sex workers] get more money if we let [clients] do it without condom, that put us at more risk of getting HIV/HPV and other diseases!”* (FGD # 2, 21-year-old, male sex worker).

### Low-risk perception to HPV infection and anal cancer

Although, participants had difficulty associating HPV infection with anal warts and sometimes warts were confused with other anal problems such as hemorrhoids. However, after discussing Ali´s vignette, participants were not only surprised to learn that nearly everyone who is sexually active will be exposed to HPV in their lifetime, but that also helped them estimating their self-risk for HPV. In addition, participants had never heard of anal cancer, the link between anal cancer and HPV infection or the need to screen for both.

“*I am very scared of it [HPV]. I wasn’t this scared before, but now, I am very scared, I am worried as I am a gay like Ali [in the vignette]!! Here we think it [STI] is not a disease of the Muslims and why is its vaccination being done? [They] are destroying us, [we] are unable to access health care facilities”* (FGD # 1, 24-year-old, MSM).

Furthermore, MSM recognized their increased susceptibility to HIV, and after learning from the Ali’s story (vignette) the similar route of transmission for HPV also, the participants realized that susceptibility chances may also be comparable to HPV since both diseases follow similar transmission pathways. Study participants seemed to be more aware of the implications of their risky behavior in terms of having unprotected sex with multiple sexual partners.

## Theme 2: Attitudes and beliefs towards HPV prevention

### Positive attitudes towards HPV vaccination and screening

Participants expressed a positive attitude toward HPV vaccination and screening. They believed that both vaccination and periodic anal screening were critical for their health in particular for those who were HIV positive. Having personal history of anal warts was the motivating factor towards receiving HPV vaccination. Although they might have been infected with the HPV before, however, spirits were high that vaccination could benefit them from other HPV types to which they had not yet been exposed. Moreover, peace of mind and protecting partners were other motivational reasons for HPV vaccine uptake.

*“Vaccine and screening are as if a drowning man catches a straw! I suffered warts multiple times, such a scary experience, when my partner moves on with someone else… having emotional trauma, anal warts is a huge problem, so after vaccination, I would have no fear of being thrown from the community, besides its your moral duty to save others after you save yourself!!”* (FGD # 1, 22-year-old, MSM).

Acceptability is associated with the knowledge and risk-perception, one of the participants vowed his concern as follow.

***“****Until now, we didn’t have knowledge. Since now we do, we will get them somehow. One must get them in any way. One must somehow arrange for it. Sex workers earn meager money …would be difficult for us! However, it's about [our] health, I will make adjustments in my spending to spare some money for it. After knowing so much…I will not delay it!”*

HIV infection makes MSM pay more attention to their health, one of the participants who was newly diagnosed with HIV showed his acceptance and willingness for HPV prevention.

*“I am glad that the vaccine would wards off the risks of HPV and also helps improve one’s condition if he is suffering from the disease, getting vaccinated has its own set of benefits like protection from cancer, warts and other skin diseases I can see the future generations safe and healthy*” (FGD#3, 25-years-old,, male sex worker newly diagnosed with HIV).

Informants who were sexual workers were supportive of prevention strategies because it would allow them to keep their clients, avoid losing income or being victims of violence.

*“We are unable to retain our clients, news spreads …as a result we are disowned by our guru… feeling of worthlessness… not bringing money in! so not only having lost means of bread but also being kicked out by our gurus so we lose the roof also and are on streets with no money and no food to eat….so now knowing about prevention we would be happy if this [CBO] or government can make arrangements for the prevention or even you[ME] itni bari dactor (well-known doctor) can arrange vaccine for us as a sadqa-e-Jariyah (gift of righteousness!”* (FGD # 6, 26-year-old, transgender women).

## Barriers for HPV vaccination and screening

Financial barriers were mentioned as key factors impairing access to HPV vaccination and screening. This was discussed to be especially true to poor LGBTQ people and those involved in subsistence sex work.

*“The one who has money will get them, but what about ones who can’t afford, people like us what should we do?”* (FGD # 2, 20-years-old, male sex worker).

Another important barrier mentioned was fear of discrimination by health workers due to their sexual orientation or their involvement in sex work. This perceived stigma made them uncomfortable disclosing their sexual orientation to health care providers, suggesting that they would be very selective about answering questions regarding their sexual behavior. Moreover, apathy and discourtesy from the doctor’s side may also discourage patients from attaining HPV immunization.

*“The biggest issue is that we are not socially accepted, [we] cannot openly discuss our issues with healthcare providers (HCPs) due to our social exclusion. [Our] social exclusion precludes disclosing health issues to HCPs”* (FGD # 2, 24-year-old, MSM).

*“We [Sex workers] are reprimanded and mocked by physicians for our choice of life, I [Sex worker] feel so much disrespect—I won’t go to him [doctor] again”* (FGD # 5, 19-year-old, male sex worker).

## Theme 3: Participants´ recommendations for HPV vaccination and screening.

### Need to raise awareness about HPV consequences among the LGBTQ community

The participants were unanimously of the view that we must always strive to educate and motivate others to get vaccinated and get the screening services. This will subsequently kick-start an ongoing cycle of raising HPV awareness- giving a deathblow to all the rumors and misconceptions attached with the side effects of the vaccine. Spreading the information forward to create a domino effect can help raise HPV awareness. They were of the view that CBOs and print and social media can also play a crucial role in creating awareness about essential knowledge of HPV.

*“Attempts to publicize HPV must be implemented by conducting such seminars to promote informed, healthy choices. Needless to say, this lack of awareness is responsible for the widespread, unchecked transmission from one individual to another.”* (FGD # 3, 24-year-old, MSM).

*“A day should be dedicated to HPV to help raise awareness in Pakistan. This is going to encourage a culture of preventive care!”* (FGD # 1, 26-year-old, MSM).

### Need to train health care providers:

The participants of a view that somehow disclosure of their sexuality become part of discussion in the context of requesting an STI testing. Some study participants shared stories of health care providers seeming uncomfortable or awkwardly asking questions, whereas others described situations in which they felt stigmatized or judged. One of the participants shared his views.

*“Health professionals under training should be exhorted (encouraged) to treat the patient irrespective of his sexual identity which is unfortunately not practiced. Thereafter, it creates a social stigma that establishes a communication gap during doctor-patient consultation*.” (FGD # 2, 22-year-old, MSM).

They very boldly recommended a need for more education of health care providers to reduce the perceived stigma for seeking health care services.

*“rampant homophobia makes the testing and screening for STIs difficult in our society, raised eyebrows and endless questions from the health care providers enables telling lies as this is the easy way out!…moreover doctors don’t understand unique needs of our community, there is a need to educate doctors and increase their competency to deal with LGBTQ health needs” and ‘‘I think it’s kind of the doctor’s job to make sure that you’re comfortable and speaking to them about whatever”.* (FGD # 2 26-year-old, MSM).

### Integration of HPV prevention into existing health service delivery

When asked where the services should be provided, almost all participants unanimously thought that public hospitals are poorly adapted to offer HPV preventive services as without any a political will and proper attitudes towards the LGBTQ population, the patient fails to receive any substantial treatment at these sites.

Participants prefer the community-based organizations for provision of these services because of the professional, unbiased treatment that incorporates maintaining the patient’s confidentiality and privacy. Moreover, they were of the view that these CBOs have always been at the forefront of providing myriad services in the past.

Furthermore, while overall participants expressed their mistrust for the public hospitals however, showed their acceptability for the services to be provided at STI clinics being run by National AIDS Control Program of Pakistan as a second choice. They thought that the government as is in the other parts of the world must take HPV vaccination seriously.

*“Vaccine can be added in childhood immunization schedule because don’t know the future sexual life to be tested for HPV routinely as we go or HIV every second month, injections should be free or subsidized for people of the community who are needy, i.e., free for non- affording people as we get the services for HIV or else price should be subsidized. The price should be set to 1/4*^*th*^* of what it is now.”* (FGD # 5, 23-year-old, MSM).

## Discussion

Our main findings from this theory-guided qualitative inquiry showed that our study sample had low knowledge of STIs in general and HPV in particular, as well as had low self-risk perception of contracting HPV. Moreover, cost and revealing sexual orientation to the health care provider were the main barriers for electing for the prevention services.

Up until our study participation in this study, most of the MSM and all transgender women were neither having any awareness or knowledge of HPV infection, which are generally consistent with the studies done in the US, UK Canada, Peru and different parts of Asian continent [47–53] nor were they aware about its negative heath consequences i.e., the association between HPV infection and anal warts and HPVs causal role in cancer development [48, 54–57] Moreover, since had no knowledge, and even after the participants were given standardized information about HPV, their perceived self-risk for HPV and HPV related diseases was also low i.e., that men can experience negative effects from HPV infection which is consistent with other studies [52, 58–60].

Risk perceptions are perhaps more important for attitudes and behaviors, for instance HPV vaccination and periodic anal Pap screening, that are intended to reduce a specific health threat such as anal warts and anal cancer and thus have a positive association between risk likelihood and subsequent action [61]. Once the participants were informed through vignette (Ali’s story) i.e., given knowledge, almost all participants adopted a positive and favorable attitude towards HPV prevention. Nevertheless, they indeed verbalized their concern that health care providers here [CBO] in sexual health clinics only focus on HIV and never talk about HPV that they considered as a new rather alien concept to them.

Moreover, many of the study participants related their poor awareness about HPV as STIs to the societal sex-adversity for discussion on sex-related issues in our country [62]. The matter of fact is that there isn’t any existing concept of sex education in Pakistan and is not a part of public discourse, infect, there is no room for any public debate on sexuality. Hence, rather unsurprisingly, no awareness is spread regarding sexual health and sexually transmitted diseases to the ordinary population and specifically to the minorities who are especially vulnerable. This implies a decreased concern when confronted with any sexual health issues. Indeed, the real culprit is the insufficient level of knowledge and awareness on the use of condoms and other protective measures. There is a surprisingly low level of condom knowledge, awareness i.e., it’s appropriate and accurate use, acceptance, and utilization among the commercial MSMs, thus collectively paving the way for STI and HIV, HPV transmission [40].

As suggested by others, [52, 63] we in our study also found that these men’s perceptions about their self-risk were associated with their low knowledge about anal cancer and never having had an anal examination by health care providers neither been informed about in social meetups in the CBO. Moreover, a recent scoping review [64] has reported the low perceived susceptibility to HPV infection and anal cancer among men who have sex with men, further explaining that many of the men have never heard about HPV from the service providers raising the questions about health care’s providers’ role and current practices in communicating information.

All participants expressed apprehensions, fear, worry, unease, and distress when given information that HPV is the most common sexually transmitted infection, most of them realized that they are at risk to acquire HPV as they are at risk of acquiring HIV as both share the same route of transmission. Moreover, HIV positivity increases individual self-care awareness and prompting them to seek knowledge and that increases willingness to adopt preventive behaviors [23]. In our study participants who were diagnosed with HIV showed their acceptability for adopting HPV prevention strategies, this finding once again suggests the importance of CBO staff and STI HCPs education, guidance, and encouragement to MSM especially after they have just been infected with HIV. HIV infected MSM are at many-fold high risk for HPV and its related diseases, helping them to get an active attitude towards HPV prevention is of great significance.

Of note, acquired HPV knowledge from the discussions, their estimated self-risk perception and HIV status were endorsed facilitating factors for the vaccination and periodic screening in our studied sample of MSM and transgender women which is in line with other studies being done in China and the United States [23, 65]. Previous studies have reported that individuals who are more aware of potential risks to their health are more likely to engage in self-protective behaviors [66].

However, one of the barriers being identified by our study participants was revealing one’s sexuality i.e., sexual identity or sexual orientation to a health professional to access these preventive services which is in agreement with other studies [67, 68] The healthcare of this sexual and gender minority is frequently conceded by their invisibility due to the discrimination and stigmatization within the health care environment [69]. Mostly these individuals report reluctance to disclose their gender identity or sexual orientation to health care providers [69], consequently, lack of disclosure leads to poor health outcomes [70]. Studies have reported [61, 71] that people who disclose their sexual orientation to HCPs are more willing to avail HPV prevention services. Moreover, attention should be given to the fact MSM are stigmatized when seeking treatment for STIs, given the stress of being in minority they cannot easily disclose their sexual orientation to the doctor and seek help [71]. Health professionals should devote attention to reducing MSM stigma, which is a challenging problem [71]. The staff of the CBOs working for MSM and transgender women in Pakistan can help this minority eliminate this shame through peer education, that in turn can increase this sexual and gender minority’s trust in the doctors. Additionally, non-disclosure may be related to the lack of sexual health competency of health care providers, HCP judgmental attitude and the fear of encountering unethical doctors [72, 73].

Consistent with other studies [50, 74–78], our study also found cost as a perceived practical barrier for employers with low wages, the sole breadwinners of the family, and for sex workers who are indeed forced into this field out of financial circumstances Similarly, the cost of the HPV preventive injections raised concerns among the middle-class population due to affordability issues. The challenge to financially manage the entire course of the vaccine independently explains why the acceptance is lagging particularly in the lower and middle-income class earning meager or insufficient daily wages [76]. As such, these people can look forward to the financial assistance extended by big well-known NGOs in Pakistan. However, when given the evidence on HPV-associated anal, penile and oral cancer risk for this minority group, most participants became interested in vaccination. Among the study participants, MSM and transgender women were more willing to be vaccinated for HPV and asked multiple questions regarding its safety and side effects [67].

Moreover, study participants expressed frustration and disappointment about the inability to get screened for HPV neither in the CBO nor is any set-up in any of the health care center by the government. This suggests that this minority group is receptive to HPV prevention and recommended to have the preventive services including anal Pap screening made available either in CBO or should be integrated in the HIV care and treatment program keeping the convenience and respect for their integrity and confidentiality in mind. To fit less educated targeted members of the community, another recommendation from the participants was to make the promotion material simple i.e., avoiding technical terms for greater understanding and reception.

### Strengths and limitations

To the best of our knowledge this is the first study on this understudied, underserved priority population on the relatively naive phenomena of HPV. This qualitative inquiry provided a more nuanced picture of the issue and explored this group’s thoughts about HPV and its preventive strategies in a more microscopic manner. Trustworthiness of the findings was ensured by PI (ME) during data collection process who spent lengthy periods of time with the study participants and maintained prolonged and trustable engagement with them to build trust so that to have a grasp of the true reality of the issue. Moreover, during entire data analysis the discussions of the findings by ME with MS was an ongoing process to enhance the credibility of the study findings. Furthermore, ME maintained a log of reflexive notes after every FDG to become aware of personal biases, personal feelings, and pre-conceived notions. Throughout the data collection process in each FGD, the PI (ME) used to share with the participants the summary statements at the end of each point before moving to the next point of discussion. Moreover, at the end of each FGD, PI (ME) conducted a wrap-up session with the recruited participants whereby summarization of the entire discussion was done to have participants validation, in order to minimize the potential for researcher bias by actively involving participants in checking and confirming the salient features of the discussion. However, our study indeed has few limitations also. Although forty-eight members from sexual and gender minority participated in the six focus group discussions, we believe that we reached the point of saturation as no new comments, ideas or themes were generated. Secondly, only a few study participants were more involved or vocal in the discussions than others; however, we tried our best to encourage others who were quiet to share their thoughts and views also. Moreover, most of our study participants were poor and had no formal education which might lead to difference in response regarding knowledge attitudes and practices. Of note, this study was conducted in an urban city in which most participants were residents. Consequently, our study results may not be applicable to those living in rural areas.

## Conclusion

The findings from this study can help public health policy and researchers to understand MSM and transgender women’s perspective on HPV prevention. The demonstrated lack of appropriate knowledge of HPV infection in this study may potentially influence the priority populations’ perception of the need to adopt preventive behaviors which are often influenced by the perceived vulnerability and severity of a condition. These findings could provide the focus points towards building culturally relevant HPV prevention interventions including HPV awareness through social and print media and CBO promotions more specifically for sexual and gender minorities.

## Recommandation

Attempts to publicize HPV must be implemented by conducting seminars to promote informed, healthy choices. Moreover, health care providers need to widen their clinical lens so that it focuses not only on the diagnosis and treatment of illness but also on health promotion of sexual and gender minority. The Pakistani government could promote a policy to pay for the vaccination of population of sexual minorities as is already covering HIV care and treatment. Creative use of technology, bundling of HPV preventive services with other types of health visits, i.e., for HIV infected MSM and transgender women, HIV clinics would be a good starting point for promoting HPV knowledge and prevention. Moreover, strategies to initiate anal Pap screening will need to improve knowledge and understanding of risk for anal cancer in this at-risk group.

## Supplementary Information

**Additional  file 1.** FGDs Interview guide

**Additional  file 2.** Ali's Story

## Data Availability

The data that support the findings of this study are available from Global & Sexual Health Research Group of Department of Global Public Health on reasonable request and with permission of Karolinska Institutet (KI) Stockholm Sweden. Dr Muslima Ejaz can be contacted on the following email addresses. Muslima.ejaz@ki.se Muslima.ejaz@aku.edu
