# Supplementary material for: Human Papillomavirus associated prevention: knowledge, attitudes, and perceived risks among men who have sex with men and transgender women in Pakistan: a qualitative study
Source: BMC Public Health. 2022 Feb 22;22:378. doi: 10.1186/s12889-022-12775-z (PMC8864907; doi:10.1186/s12889-022-12775-z)
Supplement: Supplementary file 1 — Additional file 1. FGDs Interview guide [file 12889_2022_12775_MOESM1_ESM.docx]

**Supplementary material 1**

FGDs INTERVIEW GUIDE

**A: HPV Knowledge**

1) What do you know about Sexually transmitted diseases?

2) Have you ever heard of human papillomavirus (or HPV)?

3) What do you know about HPV (Human Papillomavirus)?

4) How did you first hear about HPV? What sources would you trust?

5) Have you ever heard of a vaccine to prevent HPV? (If yes, what have you heard?)

6) Where did you hear about the HPV vaccine? (Probe: Which of these sources [repeat/display

response to previous question] would you trust the [most/least] to provide information about HPV?

**We will show HPV Vignette** (containing story with pictures)

What stands out or surprises you? What else would you like to know?

a. Based on what you know now about HPV, what do you think your risk is of [having been/becoming] infected with HPV? Or developing an HPV related disease?

**B: Determinants of intention towards HPV vaccination.**

**Perceptions about their**;

1. **Vulnerability/susceptibility/self-risk:** Based on what you know now about HPV, what do you think your risk is of [having been/becoming] infected with HPV? Or developing an HPV related disease?

- Do you think that you will get genital HPV infection in the future?
  - Probe……. if yes, how, if no, why not?
- Do you think that you will get genital warts from HPV?
  - Probe……. if yes, how, if no, why not?
- Do you think that you will get HPV related anal cancer at some point in your life?
  - Probe……If yes, how, if no, why not?

1. **Benefits/attitudes:** What about the benefits of getting treatment for the illness? have you ever considered that?

- Probes:.
  - The HPV vaccine will protect people from getting genital HPV
  - The HPV vaccine will be effective in preventing genital HPV infection
  - The HPV vaccine will reduce a man’s chances of developing HPV related cancers
  - The benefits of getting the HPV vaccine outweigh the potential risks
  - Getting the HPV vaccine may be a good thing to do for your health
  - Getting the HPV vaccine will give you a peace of mind about your health.
  - Getting the HPV vaccine will help you prevent getting anal cancer.
  - Getting the HPV vaccine will help you prevent getting genital warts.
- What do you think that in general, people who are important to you might encourage you to receive the HPV vaccine?
  - Probe…… can think *of more than one person in your life* who would like for you to get the HPV vaccine?
  - Probe…… can think of *at least one important person in your life* who would encourage you to get the HPV vaccine?
- What if the vaccine is made available to you would you like to take?

Probes

- - Whether or not you get the HPV vaccine is not up to you.
  - There are other factors beyond your control that would prevent you from getting the HPV vaccine

1. **Barriers:** Had you thought of any problems of accessing treatment for the disease? (Perceived barriers)

Probes:

- What about the cost? Having to pay a lot for the vaccine.
- Fear that you could get HPV from getting the vaccine.
- Having to get shots.
- Fear that people might think poorly of you if you got vaccinated for HPV.
- Worrying that the vaccination won’t work.
- Worrying that the vaccine isn’t safe.
- If government wouldn’t pay for the vaccine?
- If it took a lot of effort to get the vaccine?
- If it took a lot of time to get vaccinated
- If I had to discuss my sexual behavior with my health care provider
