# Supplementary material for: Human Papillomavirus associated prevention: knowledge, attitudes, and perceived risks among men who have sex with men and transgender women in Pakistan: a qualitative study
Source: BMC Public Health. 2022 Feb 22;22:378. doi: 10.1186/s12889-022-12775-z (PMC8864907; doi:10.1186/s12889-022-12775-z)
Supplement: Supplementary file 2 — Additional file 2. Ali's Story [file 12889_2022_12775_MOESM2_ESM.docx]

**Ali’s story**

| 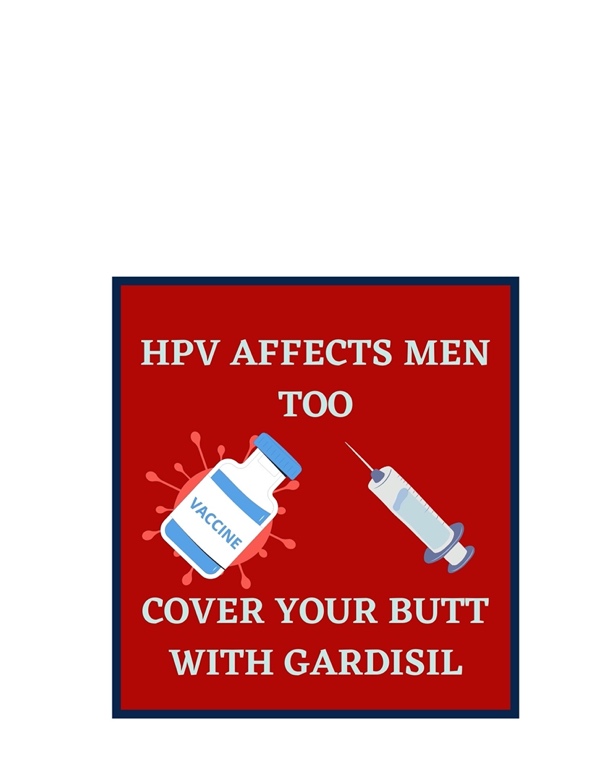Ali is a young gentleman in his early twenties. He works as a male sex worker and meets most of his clients at parties. He keeps fit by exercising regularly and tries to watch what he eats. He does not have any medical problems and so rarely visits a doctor, however, considering his profession, he tries to get tested for STIs on regular intervals. Outside of his work he enjoys spending time with his friends and family.  Recently, after a long week, Ali made plans to have dinner with his friends to unwind and relax. They planned to go to a restaurant just outside of the city. Before the dinner, he had to visit the STI clinic to give samples for his routine tests. The clinic had been renovated since Ali last visited and there were multiple fliers all around the waiting area. He went and glanced at all the posters, he was familiar with most of the diseases mentioned, however, he came across a poster which caught his attention. It was about the disease HPV. It read…….  ***“Get yourself screened and vaccinated today…. HPV is a preventable disease”***  Under this tagline, there was information about the disease. Ali read it with a lot of interest since he was not aware of this disease.   \| **WHAT is HPV?** \| **WHY you should care?** \| \| --- \| --- \| \| - HPV is a sexually transmitted virus that you can get from skin-to skin contact during all types of sexual activities - Most people will become infected with HPV in their lifetime - Most of the time HPV is asymptomatic and hence, people don’t know they have it - 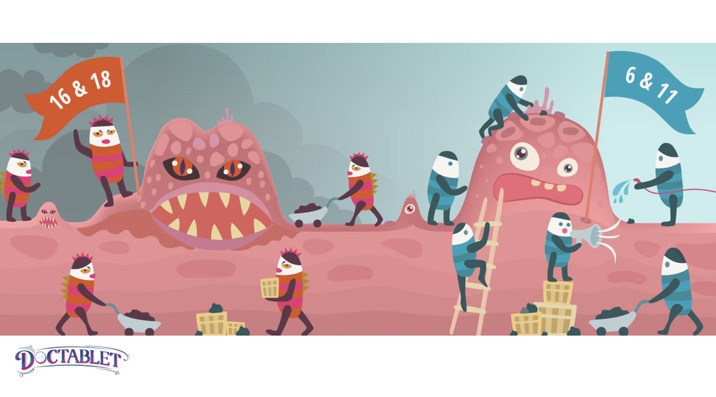 \| - For most people, HPV is self-limiting but in some cases in can progress and cause genital and anal warts. Moreover, it can cause some types of cancers including anal cancer. - Men who have sex with men are 17 times more likely to develop anal cancer than other men \| \| **HOW to prevent HPV?** \| \| \| - GARDASIL – The HPV vaccine – protects against genital warts and cancers caused by HPV - Gardasil is protective against HPV strains 6, 11, 16 and 18 - HPV vaccine is recommended for all people, 26 years old and younger   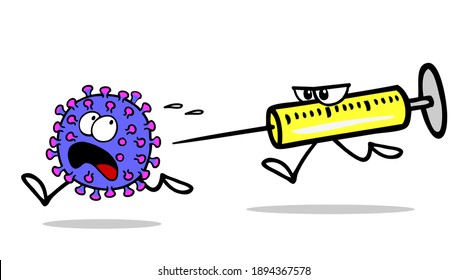  11  16  18  6 \| \| \|  \| \| \| As soon as Ali read the poster, he started feeling anxious and got worried. He was not aware of the fact that HPV can be contracted through sexual contact and may also progress and become cancerous. He had never gotten tested for the disease and was worried he might have it since he was in the high-risk groups. As he was just thinking about the possibility of him having the disease, his name was called for sample collection. He then left the clinic not paying much heed to the poster as he wanted to enjoy a great evening with his friends. \| \| \| Ali thoroughly enjoyed his hangout, came home late and slept as soon as he reached. He woke up the following day with this worrying and upsetting thought about him being HPV positive running through his mind, he started searching multiple sites on the internet and learned more about the disease, its treatment and prevention.  He found out that HPV has multiple subtypes and some subtypes are more dangerous than the other. He also found out ways to diagnose it and treat the lesions if one does get it. The most valuable thing he discovered was that it has a vaccine available against a few strains of the virus and getting the completed course of the vaccine before the age of 26 would be protective against 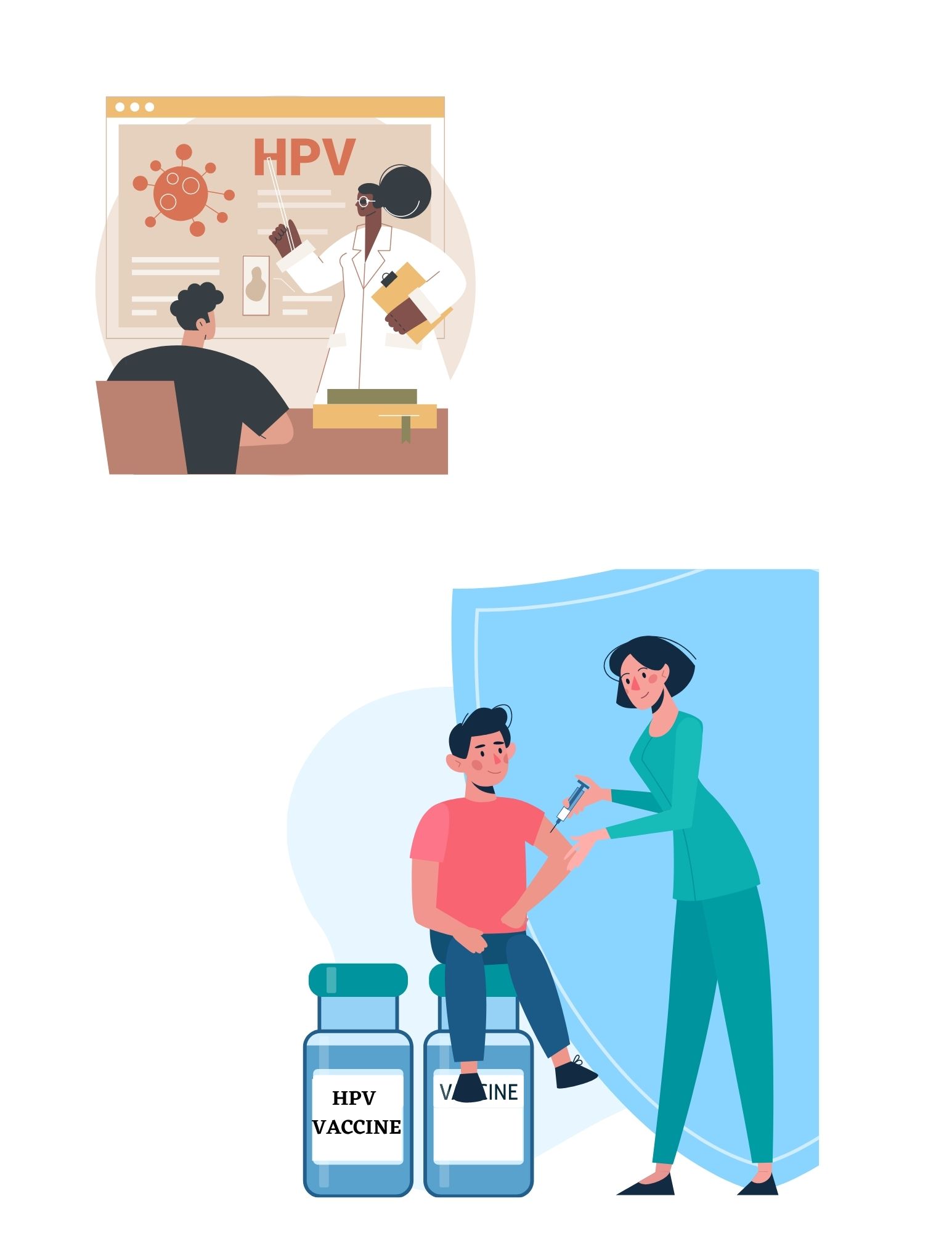HPV. \| \|  \| 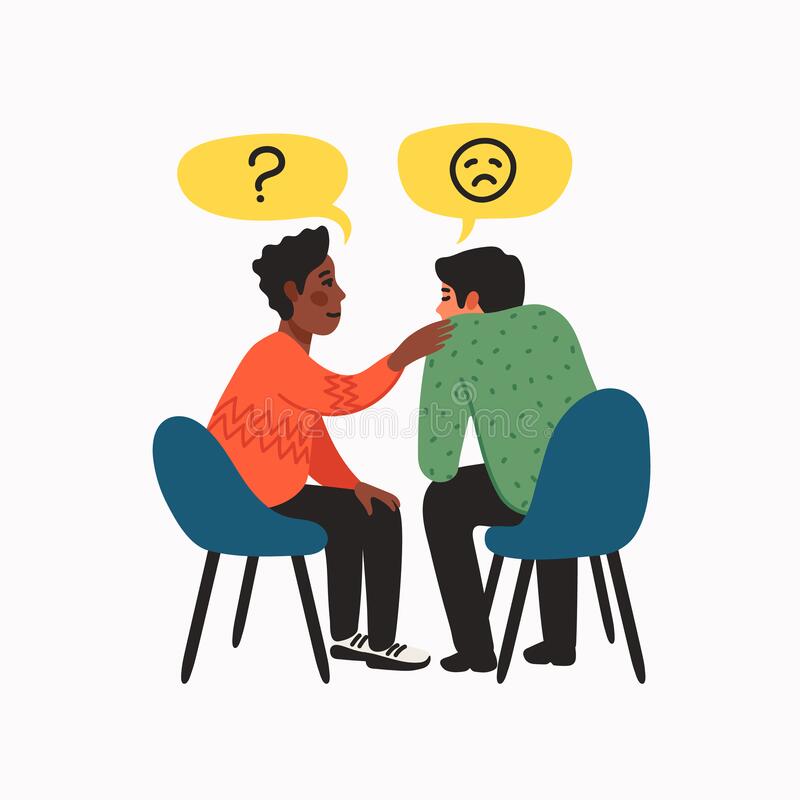Ali got busy with his work and some other personal commitments, he forgot all about the disease, the possibility of him having it and about the vaccine  After a few weeks, Ali met with his best friend Ahmed for evening tea. They were just catching up when Ahmed mentioned that he recently visited a doctor for multiple outgrowths he had developed around his anal region. He mentioned they were extremely itchy and tender, so much so that Ahmed would not be able to sit or sleep properly. Ahmed went to his local doctor for it who examined Ahmed and told him he had anal warts.  Luckily for Ahmed, the warts were still on the external surface of the body and the doctor performed a minor day care procedure, removing them without Ahmed having to go through any major surgery. Even though Ahmed’s wart had been removed and he was not experiencing any pain, he was worried that they might recur as HPV is not curable.  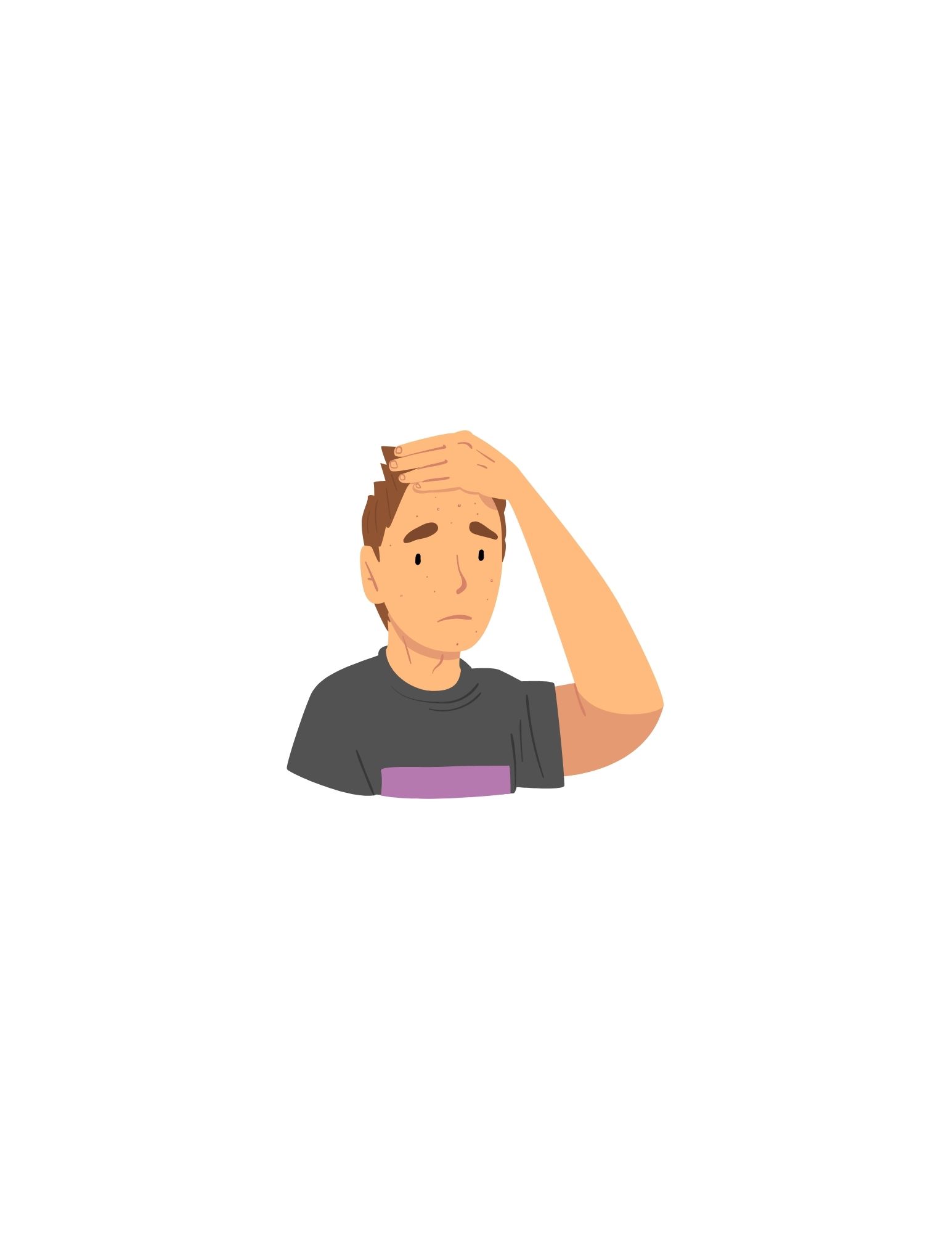Ali put on a brave face and reassured Ahmed that he shouldn’t worry and they would not reappear if he followed what the doctor had recommended, but deep-down Ali started to feel anxious and panicked for his own self.  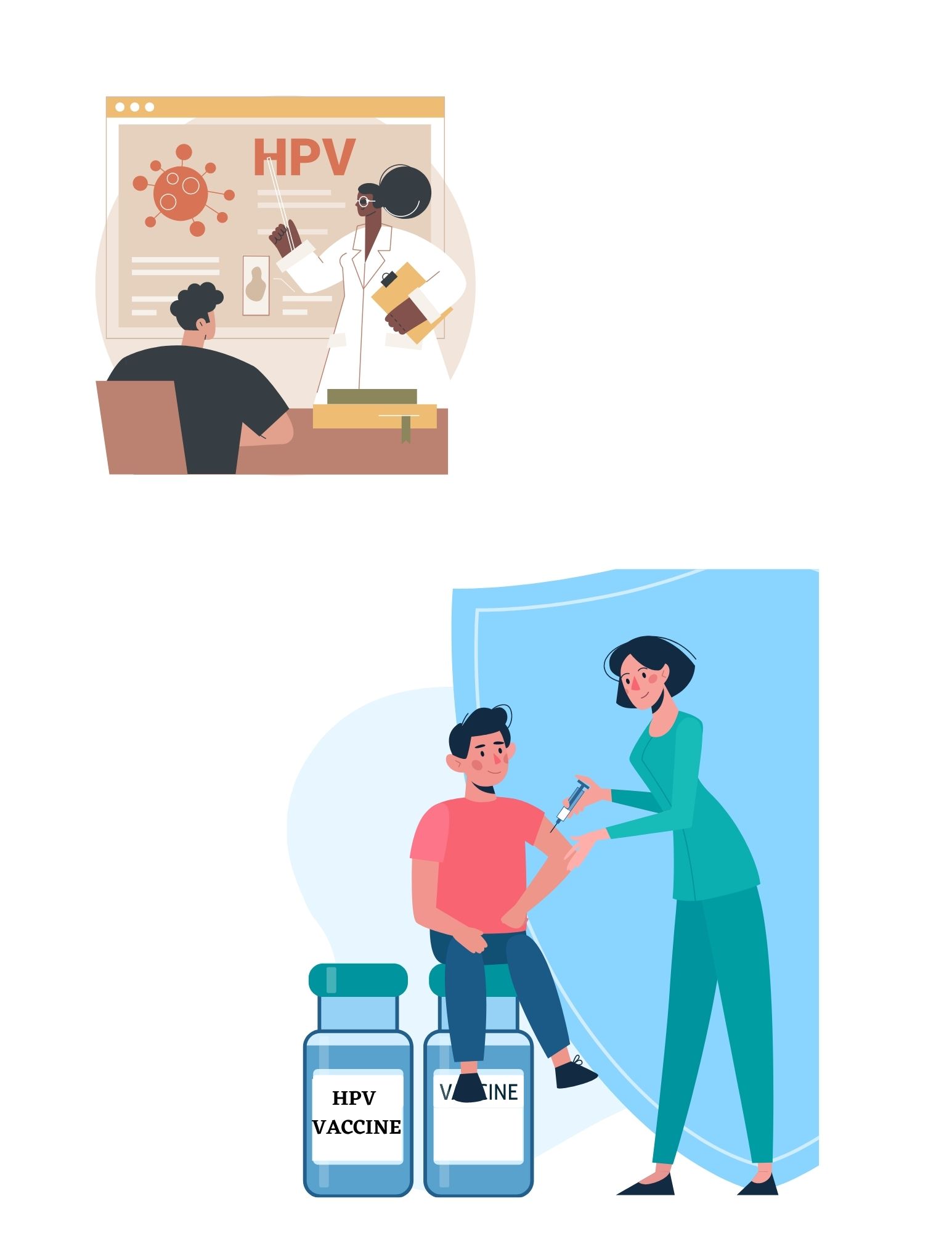After meeting with Ahmed and listening to his horrible and traumatising experience, Ali decided to get the HPV vaccine and scheduled the appointment for the next day to get his first dose. \| \| --- \|   What do you think ALI should do?  What would you do if you were in Ali’s situation?  What do you think about Ali’s decision?  Would you have decided to do the same thing (i.e., decide to get the HPV vaccine)?  Where would you go to get the HPV vaccine if you were in Ali’s situation?  Probe: HIV testing site; pharmacy, Local GP; CBOs working for gays, bisexual or transgender  Ali wants to get the HPV vaccine; he is worried that the vaccine is too expensive. What would you think of the cost of the vaccine, would the cost holdback your decision to be vaccinated?  What do you think who can influence Ali’s decision to be vaccinated? |
| --- | --- | --- | --- | --- | --- | --- | --- | --- | --- | --- | --- | --- | --- | --- | --- |
